# Obtaining EQ-5D-3L utility index from the health status scale of traditional Chinese medicine (TCM-HSS) based on a mapping study

**DOI:** 10.1186/s12955-022-02076-9

**Published:** 2022-12-15

**Authors:** Li Wang, Yuqiong Lu, Zhanjing Dai, Penghua Shi, Jiayi Xu, Feng Chang, Yun Lu

**Affiliations:** Center for Health Care Policy Research, School of International Pharmaceutical Business, China Pharmaceutical University, 639 Longmian Avenue, Jiangning District, Nanjing, 211198 Jiangsu China

**Keywords:** Mapping, Utility, TCM-HSS, EQ-5D-3L, Quality of life measures

## Abstract

**Background:**

Almost all traditional Chinese medicine (TCM) quality of life measures are non-preference-based measures (non-PBMs), which do not provide utilities for cost-utility analysis in pharmacoeconomic evaluation. Whereas the mapping has become a new instrument to obtain utilities, which builds a bridge between non-PBMs and PBMs.

**Purpose:**

To develop mapping algorithms from the health status scale of traditional Chinese medicine (TCM-HSS) onto the three-level EuroQol five-dimensional questionnaire (EQ-5D-3L).

**Methods:**

The cross-sectional data were collected by questionnaire survey from a tertiary hospital visit population and community residents in China, and randomly divided into training and validation set by 2:1. Based on the training set, direct and indirect mapping methods (7 regression methods and 4 model specifications) were conducted to establish alternative models, which were comprehensively evaluated based on the validation set by mean absolute error, root mean square error, and Spearman correlation coefficient between predicted and observed values. Based on the whole sample, the preferred mapping algorithm was developed.

**Results:**

A total of 639 samples were included, with an average age of 45.24 years and 61.66% of respondents were female. The mean EQ-5D-3L index was 0.9225 [SD = 0.1458], and the mean TCM-HSS index was 3.4144 [SD = 3.1154]. The final mapping algorithm was a two-part regression model including the TCM-HSS subscales, interaction terms, and demographic covariates (age and gender). The prediction performance was good. The mean error was 0.0003, the mean absolute error was 0.0566, the root mean square error was 0.1039, and 83.10% of the prediction errors were within 0.1; the Spearman correlation coefficient between predicted and observed EQ-5D-3L values was 0.6479.

**Conclusion:**

It is the first study to develop a mapping algorithm between the TCM-HSS and EQ-5D-3L, which demonstrates excellent prediction accuracy and estimates utility value for economic evaluation from TCM quality of life measures.

**Supplementary Information:**

The online version contains supplementary material available at 10.1186/s12955-022-02076-9.

## Introduction

Pharmacoeconomic evaluation (PE), an effective method for rational allocation of health resources, has become an important basis for governments around the world to make health decisions. The "Interim Measures for the Administration of Medication for Basic Medical Insurance" [1] published by China had clearly required companies to submit PE-related materials in the adjustment process of the medical insurance drug list. The National Medical Security Administration of China, established in 2018, had carried out four consecutive negotiations for medical insurance drug list. The proportion of Chinese patent medicines in the list is gradually equal to that of Western medicines (49.43%, 49.07% and 48.04% in the past three years). With the continuous improvement of the dynamic adjustment mechanism of Chinese medical insurance catalogue, the economic evaluation of traditional Chinese medicine (TCM) will inevitably become one of the normalized tasks, and high-quality and standardized PE evidence of TCM has become an urgent need.

Cost-utility analysis (CUA) is one of the common methods in PE and the preferred recommendation in international pharmacoeconomic guidelines [2], and the key to CUA is the measurement of health utility (HU). It is obtained indirectly through a preference-based measure that has established a utility value scoring system, such as the three-level EuroQol five-dimensional questionnaire (EQ-5D-3L), the most widely applied in the world. Nevertheless, traditional Chinese medicine quality of life measures are mostly not based on preference, lacking utility value scoring systems, and cannot measure utility value directly. Yu [3] systematically reviewed the Chinese and English databases and found that there were eight generic TCM quality of life scales, all of which were not based on preference, like the health status scale of traditional Chinese medicine (TCM-HSS), Chinese quality of life instrument (Ch QOL), health scale of traditional Chinese medicine (HSTCM), etc. Wang [4] searched Chinese databases and found that there were 39 TCM disease-specific quality of life scales, all of which were non-utility measures, such as the quality of life scale in patients with chronic hepatitis B (AOL-CHB), quality of life scale for chronic eczema patients-prior test version (EQOLS), etc. Fortunately, the mapping technique or cross-walking are recommended by the UK National Institute of Health and Clinical Excellence (NICE) [5], which provides a new idea for non-preference-based measures (non-PBMs) to obtain utility value.

The mapping method predicts the utility value of non-PBMs by building mapping algorithms between the non-PBMs (also called starting scales,) and preference-based measures (PBMs, also called target scales). However, no one has applied the mapping method to the field of TCM probably because the differences in cultural background and medical philosophy between Chinese and Western medicine may make it more difficult to make a connection between the Chinese and Western scales, thus, it is essential to conduct the first study as a reference for subsequent studies of this kind. Among the manufactured generic TCM quality of life scales, the TCM-HSS, one of non-PBMs, is the most comprehensive measure in terms of reliability and validity assessment, and the test results showed excellent reliability and validity [6]. The measure was completely available accompanied with a distinct scoring method including item, subscale and total scores provided by the developers at the same time [6]. Apart from that, the TCM-HSS had been more widely used in various studies, for instance, some scholars had used the TCM-HSS in clinical investigations of different diseases so as to assess the responsiveness of TCM-HSS to different disease types, e.g., Zeng [7, 8]conducted clinical investigation on patients with four diseases (coronary heart disease, rheumatoid arthritis, chronic obstructive pulmonary disease, chronic renal failure) by TCM-HSS and SF-36, and Liu [9] used the TCM-HSS to assess changes in the health status of patients with functional gastrointestinal disease before and after treatment. Additionally, the TCM-HSS was also applied to evaluate the health status of the general population, e.g., Chen [10] adopted the TCM-HSS as a survey instrument to investigate the quality of life of college students and explored the factors affecting the TCM-HSS scores. Therefore, the aim of the study is to develop a mapping algorithm between TCM-HSS (a starting scale) and EQ-5D-3L (a target scale), so as to break the technical barriers that the TCM quality of life measures cannot obtain the utility value, improve the evidence level in the economic evaluation of traditional Chinese medicine, and promote the high-quality development of the traditional Chinese medicine industry.

## Materials and methods

### Data

The population who visited a tertiary hospital in Jiangsu Province from February to May 2021 were recruited through a combination of online and offline questionnaire survey. Additionally, an online survey of community residents was conducted during the same period. The inclusion criteria were: (1) aged 16 or above, (2) informed consent to the survey, (3) not cognitive impairment and able to complete the questionnaire independently. Participants who did not meet the inclusion criteria were excluded. Participants completed the TCM-HSS and EQ-5D-3L simultaneously. Moreover, we collected demographic and health-related information including age, gender, marital status, education level, smoking, drinking, etc. The total sample consisted of the above two sections and 23 data with serious missing information were eliminated.

### Instruments

#### EQ-5D-3L

EQ-5D-3L, a generic preference-based measure, was developed by the European Quality of Life Group [11]. It is one of the most widely used quality of life measures in the world. More than a dozen countries have developed utility value scoring systems based on the preference of their native population. The health utility scoring system of EQ-5D-3L was primarily developed by Liu et al. [12] based on the Chinese general population in 2014. The EQ-5D-3L includes 5 dimensions: mobility, self-care, usual activity, pain/discomfort, and anxiety/depression. Each dimension includes 3 levels: no difficulty, some difficulty and extreme difficulty, which can describe 3^5^ = 243 different health states.

#### TCM-HSS

TCM-HSS, a generic non-preference-based measure of TCM, was developed by Professor Liu F B 's team [13] under the guidance of the traditional Chinese medicine theory and widely used in the clinical field of TCM in China. The initial version in 2008, including 8 subscales and 30 items, was revised and improved to form the final version with 33 items in 8 subscales in 2009 [6]. Eight subscales include energy, pain, diet, stool, urination, sleeping, physical and mood, which occupy 32 items, and each subscale consists of 2–8 items. The last item is an overall evaluation of health. Each item is answered with a 4-level rating method, including very good, general, slightly poor, very poor with a score of 0 to 3 points, and the lower score indicates the better quality of life. The score of each subscale of the scale = sum of the scores of the items belong to the subscale/the number of items belong to the subscale, and the total scores of the scale = sum of the scores of all items. We had delivered a table comparing the structural components of the TCM-HSS and EQ-5D-3L, please see Additional file 1: Table S1.

### Statistical methods

The correlations were calculated to assess the degree of conceptual overlap between the starting and target scales before the mapping process considering the different development contexts of the two scales, which was to ensure that a link could be established between TCM-HSS and EQ-5D-3L [14]. The Spearman's rank correlation was used to evaluate the association between the two measures, which denoted weak, moderate, or strong correlation for coefficient < 0.35, ≥ 0.35 and ≤ 0.5, or > 0.5 [15].

The mapping process was as follows: first, the whole sample was divided into the training set and validation set by random blocking method with age as a blocking factor according to 2:1; then, seven econometric methods and four model specifications were combined to construct alternative models based on the training set, and we evaluated the alternative models to select a good predictive performance of model specification based on the validation set; finally, based on the whole sample the final mapping algorithm was established in the model specification selected above and checked its predictive performance.

The four model specifications were described as alternative independent variables: (1) total score of TCM-HSS; (2) total score of TCM-HSS plus covariates (age and gender); (3) eight subscale scores of TCM-HSS (i.e., energy, pain subscale); (4) eight subscale scores of TCM-HSS plus covariate (age and gender).

The previous systematic reviews found that the most widely used econometric model in mapping was ordinary least square (OLS), while other methods that consider the characteristics of health utility value distribution were also applied [16, 17]. These included Tobit model [18, 19] and censored least absolute deviations (CLAD) [20, 21] to handle censored or bounded data as the maximum of utilities is 1 as well as beta regression [22] and fractional logistic model [23]. Generalized linear model (GLM) was also used, which offers flexibility in various distribution types [24]. Robust regressions in liner regressions like MM-estimator, M-estimator, etc. were also applied, which are designed to deal with the effect of outliers [25]. Furthermore, approaches which enable greater flexibility such as mixture models, which encompass multiple models have been developed. So do the two- or three-part models (TPM or 3PM), which combine logistic and liner regression models for those who are at and below the full health. Methods like adjusted limited dependent variable mixture model (ALDVMM), developed for EQ-5D data specially, which combined consideration of potential distribution of data and a limit of utility values [26, 27], were increasingly applied in mapping studies. Techniques such as multinomial logistic regression (MLR) or ordinal logistic regression model, which are suitable for disordered or ordered multi-classification dependent variables, respectively, had been used for indirect mapping to PBM dimensions or items.

In our study, seven econometric methods were conducted including OLS, Tobit, CLAD, GLM, ALDVMM, TPM and ordinal logistic regression. All methods except the last one, an indirect or response mapping method, are direct mapping methods, which directly predict the EQ-5D-3L score (based on the Chinese tariff [12]). We considered the negative utility value (1 minus EQ-5D-3L utility value) as the dependent variable, and the specified distribution family was gamma when using GLM. ALDVMM was set to 2 components after the model failed to converge when testing with up to three components. The logit regression was adopted for the first part and an aforementioned GLM was executed for the second part in TPM [28], and the expected utility was estimated as Pr (Utility = 1) + U* (1-Pr (Utility = 1)) [U: predicted utility at imperfect health; Pr (Utility = 1): predicted probability at full health]. In response mapping, which predicted the response level of five questions in EQ-5D, and then calculated the EQ-5D-3L utility value according to health utility scoring system, we conducted the ordered logistic regression model after testing because it depends on the parallelism assumption and another choice is multinomial logistic regression if violating the assumption [29, 30].

We assessed the predictive performance of alternative models based on the comprehensive consideration of three indicators: (1) mean absolute error (MAE), the smaller, the better; (2) root mean squared error (RMSE), the smaller, the better; (3) Spearman's rank correlation coefficient between the observed and predicted EQ-5D-3L values, the closer is to 1, the better. All statistical analyses were performed in Microsoft Excel and Stata/MP (Stata Corp. LP, College Station, Texas). The manuscript followed the mapping onto preference-based measures reporting standards (MAPS) checklist [31] (see Additional file 2).

## Results

### Descriptive summary

639 respondents were included in the analysis eventually, of which 581 were derived from the hospital visit population and 58 from community residents. The average age of respondents was 45.24 years old with 61.66% being female. More than half of the sample were employed (52.9%) and sometimes controlled their diet (52.11%). Most of them were married (71.52%), never smoked (77.46%), never drank (59.62%), and participated in basic medical insurance for employees (63.07%). More specific information is shown in Table 1.

**Table 1:** Demographics characteristics of the sample

Variance	Overall N (%)	Training^#^ N_1_ (%)	Validation^#^ (%) N_2_ (%)
N (%)	639 (100)	426 (66.67)	213 (33.33)
Age-mean (SD)	45.24 (18.26)	45.09 (18.3)	45.54 (18.22)
BMI-mean (SD)	23.23 (3.46)	23.15 (3.36)	23.38 (3.66)
Gender			
Male	245 (38.34)	164 (38.50)	81 (38.03)
Female	394 (61.66)	262 (61.50)	132 (61.97)
Consultation			
Clinic	356 (55.71)	234 (54.93)	122 (57.28)
Hospitalized	283 (44.29)	192 (45.07)	91 (42.72)
Smoking			
Never	495 (77.46)	327 (76.76)	168 (78.87)
Occasionally	56 (8.76)	36 (8.45)	20 (9.39)
Frequently	65 (10.17)	48 (11.27)	17 (7.98)
Quit smoking	23 (3.6)	15 (3.52)	8 (3.76)
Drinking			
Never	381 (59.62)	259 (60.8)	122 (57.28)
Occasionally	206 (32.24)	133 (31.22)	73 (34.27)
Frequently	34 (5.32)	22 (5.16)	12 (5.63)
Quit drinking	18 (2.82)	12 (2.82)	6 (2.82)
Dieting			
Never	156 (24.41)	95 (22.30)	61 (28.64)
Sometimes	333 (52.11)	224 (52.58)	109 (51.17)
Frequently	150 (23.47)	107 (25.12)	43 (20.19)
Excising			
Never	125 (19.56)	84 (19.72)	41 (19.25)
Sometimes	414 (64.79)	271 (63.62)	143 (67.14)
Frequently	100 (15.65)	71 (16.67)	29 (13.62)
Employment			
Employed	338 (52.9)	224 (52.58)	114 (53.52)
Unemployed	131 (20.5)	86 (20.19)	45 (21.13)
Retirement	170 (26.6)	116 (27.23)	54 (25.35)
Insurance			
Employee	403 (63.07)	268 (62.91)	135 (63.38)
Resident	187 (29.26)	124 (29.11)	63 (29.58)
Others	30 (4.69)	22 (5.16)	8 (3.76)
No	19 (2.97)	12 (2.82)	7 (3.29)
Marriage			
Unmarried	151 (23.63)	108 (25.35)	43 (20.19)
Married	457 (71.52)	298 (69.95)	159 (74.65)
Widowed	18 (2.82)	9 (2.11)	9 (4.23)
Divorced	13 (2.03)	11 (2.58)	2 (0.94)
Income_m^$^			
< 1000	120 (18.78)	79 (18.54)	41 (19.25)
1000–4999	277 (43.35)	172 (40.38)	105 (49.30)
5000–9999	183 (28.64)	130 (30.52)	53 (24.88)
≥ 10,000	59 (9.23)	45 (10.56)	14 (6.57)
Education			
Junior or below	186 (29.11)	114 (26.76)	72 (33.80)
Senior or secondary	159 (24.88)	107 (25.12)	52 (24.41)
Tertiary	101 (15.81)	64 (15.02)	37 (17.37)
Undergraduate	158 (24.73)	114 (26.72)	44 (20.66)
Master or above	35 (5.48)	27 (6.34)	8 (3.76)

^#^Training set and validation set

^$^Income of one month

N, number of respondents; SD, standard deviation

With age as the block factor, the whole sample was randomly grouped into training set (N_1_ = 426) and validation set (N_2_ = 213) by 2:1. The basic information is also shown in Table 1. Moreover, hypothesis testing of differences between training and validation sets in terms of age, gender, etc., was performed to ensure the two subsets were balanced (see Additional file 1: Table S2). The test results showed no significant differences between the two subsets in terms of demographics (e.g., gender, age, BMI) and scale scores (*P* > 0.05).

### Characteristics of the two instruments

The mean EQ-5D-3L utility index was 0.9225 [standard deviation (SD) = 0.1458] with the minimum and the maximum value being 0.056, 1.000 respectively. The mean TCM-HSS score was 3.4144 (SD = 3.1154) with the minimum and the maximum value being 0, 19.4333 respectively. The average scores of the eight subscales in TCM-HSS were all less than 1, with the highest in energy subscale (mean = 0.6174; SD = 0.5175) and the lowest in urination subscale (mean = 0.1758; SD = 0.3115). More specific information including the training and validation set was shown in Table 2. The Spearman's rank correlation coefficients between TCM-HSS and EQ-5D-3L were all moderate to strong (*P* < 0.05), indicating that there was some connection and mapping algorithm were fitted between the two scales. The correlation results are displayed in Additional file 1: Table S3–S4 for details.

**Table 2:** Characteristics of EQ-5D-3L index and TCM-HSS score

Variance	Overall Mean (SD)	Training^#^ Mean (SD)	Validation^#^ Mean (SD)
N (%)	639 (100)	426 (66.67)	213 (33.33)
EQ-5D-3L-mean (SD)	0.9255 (0.1458)	0.9235 (0.1502)	0.9296 (0.1369)
Range of EQ-5D-3L	0.056–1.000	0.056–1.000	0.114–1.000
Distribution of EQ-5D-3L			
MO 1/2/3, %	92.18/5.63/2.19	91.55/6.34/2.11	93.43/4.23/2.35
SC 1/2/3, %	95.15/2.50/2.35	95.07/2.35/2.58	95.31/2.82/1.88
UA 1/2/3, %	92.33/5.48/2.19	91.78/5.87/2.35	93.43/4.69/1.88
PD 1/2/3, %	79.97/19.87/0.16	80.75/19.01/0.23	78.40/21.60/0
AD 1/2/3, %	82.94/16.59/0.47	82.16/17.14/0.70	84.51/15.49/0
TCM-HSS-mean (SD)	3.4144 (3.1154)	3.4278 (3.1077)	3.3875 (3.1378)
Range of TCM-HSS	0–19.4333	0–19.4333	0–15.6000
EnT	0.6174 (0.5175)	0.6288 (0.5129)	0.5945 (0.5271)
PaT	0.4460 (0.6208)	0.4613 (0.6327)	0.4155 (0.5967)
DiT	0.3089 (0.4112)	0.3103 (0.4149)	0.3061 (0.4045)
StT	0.2753 (0.4775)	0.2613 (0.4612)	0.3031 (0.5084)
UrT	0.1758 (0.3115)	0.1690 (0.3179)	0.1894 (0.2987)
SlT	0.3375 (0.5252)	0.3294 (0.5171)	0.3537 (0.5419)
PhT	0.1984 (0.3389)	0.1954 (0.3353)	0.2042 (0.3468)
MoT	0.3697 (0.4862)	0.3773 (0.5009)	0.3545 (0.4562)

EQ-5D-3L, three-level EuroQol five-dimensional questionnaire; TCM-HSS, health status scale of traditional Chinese medicine; SD, standard deviation; MO, mobility; SC, self-care; UA, usual activities; PD, pain/discomfort; AD, anxiety/depression; EnT, total scores of energetic subscale; PaT, total scores of painful subscale; DiT, total scores of dietary subscale; StT, total scores of stool subscale; UrT, total scores of urination subscale; SlT, total scores of sleeping subscale; PhT, total scores of physical subscale; MoT, total scores of mood subscale

^#^Training set and validation set

### Comparison of model performance

Each regression method included four model specifications, thus, a total of 28 candidate models were developed based on the training set. Table 3 shows the specific predictive performance of candidate models and the one with the best predicted performance has been bolded. Based on the validation set, TPM4 was the best performing model followed by ALDVMM4, and GLM2 or GLM4 was inferior. TPM4 was a regression method using a two-part model with eight subscale scores in TCM-HSS and covariates (age and gender) as independent variables in line with comprehensive ranking of the three indicators. The MAE, RMSE, and Spearman's rank correlation coefficient between the observed and predicted EQ-5D-3L values in the TPM4 model were 0.0554, 0.0974, and 0.6550, respectively. Besides, the 28 alternative models to predict overestimation or underestimation of EQ-5D-3L utility values were also computed based on the validation set. The results are detailed in Additional file 1: Table S5-S6.

**Table 3:** Predictive performance evaluation of alternative models (N_2_ = 213^#^)

	MAE	RMSE	Rho	Rank MAE	Rank RMSE	Rank Rho	Final rank
OLS1	0.0627	0.1032	0.6354	15	10	15	12
OLS2	0.0638	0.1002	0.6351	16	6	21	14
OLS3	0.0606	0.1036	0.6454	13	11	10	9
OLS4	0.0613	0.1009	0.6468	14	7	9	7
Tobit1	0.2211	0.2511	0.6354	28	23	15	25
Tobit2	0.2181	0.2477	0.6367	25	21	13	23
Tobit3	0.2202	0.2514	0.6508	27	24	5	22
Tobit4	0.2189	0.2500	0.6555	26	22	1	18
CLAD1	0.0891	0.1218	0.6354	19	19	15	21
CLAD2	0.0861	0.1205	0.6373	18	17	12	17
CLAD3	0.0797	0.1157	0.6541	17	14	3	9
CLAD4	0.1531	0.1849	0.6470	24	20	8	19
GLM1	0.1075	0.3654	0.6354	20	25	15	24
GLM2	0.1264	0.4873	0.6313	23	28	22	27
GLM3	0.1196	0.4606	0.6283	21	26	23	26
GLM4	0.1231	0.4822	0.6235	22	27	24	27
TPM1	0.0585	0.1015	0.6354	12	8	15	11
TPM2	0.0563	0.0947	0.6377	6	2	11	3
TPM3	0.0571	0.1021	0.6479	10	9	7	6
**TPM4**	**0.0554**	**0.0974**	**0.6550**	**4**	**3**	**2**	**1**
ALDVMM1	0.0580	0.0987	0.6354	11	4	15	7
ALDVMM2	0.0570	0.0941	0.6361	9	1	14	4
ALDVMM3	0.0568	0.1037	0.6505	7	12	6	5
ALDVMM4	0.0554	0.0996	0.6517	5	5	4	2
OLOGIT1	0.0570	0.1196	0.4368	8	16	28	19
OLOGIT2	0.0535	0.1112	0.4720	3	13	27	14
OLOGIT3	0.0513	0.1213	0.4894	2	18	26	16
OLOGIT4	0.0499	0.1158	0.5363	1	15	25	13

OLS, ordinary least square; CLAD, Censored least absolute deviations; GLM, generalized linear model; ALDVMM, adjusted limited dependent variable mixture model; TPM, two-part model; OLOGIT, ordinal logistic regression; MAE, mean absolute error; RMSE, root mean squared error; Rho, Spearman's rank correlation coefficients;1–4, four model specifications

^#^Validation set

### Establishment and evaluation of the final mapping algorithm

The coefficients of variables for the final mapping algorithm were obtained from the whole sample. Square terms and interaction terms, perhaps to improve performance in the final mapping algorithm, were taken into account as in other studies [32, 33]. Thus, we took four steps to make adjustments for the final mapping model as follows. (1) A two-part regression model with the TCM-HSS eight subscales scores as independent variables was performed based on the total sample; (2) The square terms (i.e., EnT*EnT, PaT*PaT, etc.), as new independent variables based on step 1, were joined and eliminated if not significant (*P* > 0.05); (3) The two-way interaction terms (i.e., EnT*PaT, EnT*DiT, etc.), as new independent variables based on step 2, were joined and excluded if not significant (*P* > 0.05); (4) Age and gender were added as new demographic independent variables based on step 3. We presented the fitting results of each step in Additional file 1: Table S7 and found that the regression model obtained in step 4 was the top performer based on the above three indicators. The variables coefficients, t-statistics and other parameters of the final mapping algorithm are listed in Table 4.

**Table 4:** The final mapping algorithm

	Coefficient	*t*	*P*	SE	95%-LL	95%-UL
logit						
EnT	0.3972	1.09	0.2768	0.3652	− 0.3186	1.1130
PaT	1.4093***	6.61	0.0000	0.2133	0.9913	1.8274
DiT	0.3034	0.80	0.4210	0.3771	− 0.4357	1.0426
StT	0.0809	0.17	0.8681	0.4871	− 0.8737	1.0355
UrT	1.9813**	3.17	0.0015	0.6256	0.7551	3.2074
SlT	0.1797	0.74	0.4582	0.2423	− 0.2951	0.6545
PhT	1.1546**	2.63	0.0087	0.4398	0.2927	2.0165
MoT	1.6874***	4.51	0.0000	0.3741	0.9541	2.4207
StTUrT	− 1.1902	− 1.79	0.0738	0.6656	− 2.4948	0.1144
UrTMoT	− 2.0671***	− 3.30	0.0010	0.6261	− 3.2942	− 0.8399
EnTStT	0.7849	1.50	0.1342	0.5240	− 0.2422	1.8120
Age	0.0166*	2.33	0.0200	0.0071	0.0026	0.0305
1.Gender	− 0.2241	− 0.87	0.3840	0.2575	− 0.7288	0.2805
_cons	− 3.6722***	− 8.09	0.0000	0.4538	− 4.5615	− 2.7828
glm						
EnT	0.0655	0.46	0.6420	0.1409	− 0.2107	0.3417
PaT	0.1072	1.47	0.1407	0.0728	− 0.0354	0.2499
DiT	0.1133	0.88	0.3811	0.1293	− 0.1402	0.3667
StT	− 0.1838	− 1.01	0.3145	0.1828	− 0.5421	0.1744
UrT	0.2562	1.13	0.2584	0.2267	− 0.1881	0.7004
SlT	− 0.0592	− 0.66	0.5065	0.0891	− 0.2338	0.1154
PhT	− 0.0108	− 0.08	0.9392	0.1414	− 0.2880	0.2664
MoT	− 0.0591	− 0.49	0.6276	0.1219	− 0.2981	0.1798
StTUrT	− 0.0690	− 0.33	0.7451	0.2124	− 0.4853	0.3472
UrTMoT	0.0706	0.34	0.7309	0.2053	− 0.3318	0.4730
EnTStT	0.2709	1.87	0.0614	0.1448	− 0.0129	0.5547
Age	0.0045	1.63	0.1029	0.0028	− 0.0009	0.0100
1.Gender	− 0.1093	− 1.03	0.3018	0.1059	− 0.3168	0.0982
_cons	− 2.0378***	− 11.23	0.0000	0.1814	− 2.3934	− 1.6822

*P < 0.05; **P < 0.01; ***P < 0.001

1.Gender, female; t, t statistics; *P*, *P*-values; SE, standard error; 95% CI, 95% confidence intervals; LL, lower limit of 95% CI; UL, upper limit of 95% CI; StTUrT, StT*UrT; UrTMoT, UrT*MoT; EnTStT, EnT*StT

In the final mapping algorithm, the prediction range is 0.2567–0.9965, and the observation range is 0.056–1.000. Nonetheless, both of them had the highest proportion in the fractional segment of (0.80, 1]. The Spearman rank sum correlation test was performed between the two, and the result was 0.6479 (*P* < 0.0001, *P* < 0.05), showing a medium–high strength correlation. We also drew a scatterplot between observed and predicted values (see Additional file 1: Fig. 1). Foreign researchers found that MAE, RMSE and mean error (ME) among the published mapping algorithms were at [0.0011, 0.19], [0.084, 0.2] and [0.0007, 0.042], respectively [16]. In our study, the ratio of AE > 0.05, ME, MAE, RMSE, and Spearman's rank correlation coefficient between the observed and predicted EQ-5D-3L values were 34.12%, 0.0003, 0.0566, 0.1039, and 0.6479, respectively (Table 5), which are within the above-mentioned scope and are at a high level. Furthermore, MAE, RMSE, and ME were calculated in different fractions (Table 5), and a line chart of the above indicators was plotted (see Additional file 1: Fig. 2).

**Table 5:** Performance evaluation of the final mapping algorithm

Observations	Frequency	AE > 0.05 (%)	AE > 0.1 (%)	MAE	RMSE	ME
Overall	639	218 (34.12%)	108 (16.90%)	0.0566	0.1039	0.0003
≤ 0.2	4	4 (100.00%)	4 (100.00%)	0.4550	0.5435	0.4550
(0.20, 0.40]	11	11 (100.00%)	11 (100.00%)	0.4197	0.4412	0.4197
(0.40, 0.60]	9	9 (100.00%)	6 (66.67%)	0.1967	0.2411	0.1967
(0.60, 0.80]	63	40 (63.49%)	28 (44.44%)	0.1006	0.1347	0.0514
(0.80, 1.00)	127	68 (53.54%)	28 (22.05%)	0.0603	0.0725	0.0211
1.00	425	86 (20.24%)	31 (7.29%)	0.0328	0.0542	− 0.0328

AE, absolute error; MAE, mean absolute error; RMSE, root mean squared error; ME, mean error

## Discussion

In this study, 28 candidate models were built by direct and indirect mapping methods first, and then the preferred model was selected based on MAE, RMSE and correlation coefficient between the observed and predicted EQ-5D-3L values. Afterwards, we adjusted the mapping model by adding squared items and interaction terms and eliminated insignificant terms (*P* > 0.05) to obtain the final mapping algorithm, a two-part regression model with eight subscale scores in TCM-HSS, three interaction terms, and covariates (age, gender) as independent variables (see Additional file 1: Table S7). Generally, the mapping algorithms tend to overestimate poorer health status and underestimate better health status [34]. We do the same, the predicted values were all below 1 but very close to 1 for the observed utilities at 1, however, the predictions were overestimated for the observed utilities below 0.6. More specific information is presented in Additional file 1: Table S8 and Fig. 3. Nevertheless, compared with the previous mapping algorithms, MAE, RMSE and correlation in our algorithm were superior, indicating good prediction accuracy.

The empirical research based on the mapping method in China is still in its infancy now. We retrieved Chinese databases (CNKI, Wan Fang and VIP), with keywords such as "mapping", "utility", "health utility", and "quality of life scale", and found that there were 17 Chinese literatures related to the mapping method in measuring utility, of which only six were empirical literatures [35–40]. Merely one of the six studies performed both direct and indirect mapping methods (a total of seven regression methods) to map EORTC QLQ-BR53 to EQ-5D-5L and SF-6D respectively [38]; while the rest conducted exclusively direct mapping method, with few methodologies.

However, the foreign empirical researches based on the mapping method to obtain utility have reached a mature stage, involving various measures and regression methods. Mukuria [17] reviewed 180 empirical mapping literatures with seven PBMs like EQ-5D as the target scales from January 2007 to October 2018, including 233 mapping algorithms and involving numerous methods like two-part/three-part models, mixed models, fractional response models, adjusted limited dependent variable mixture model, etc. Notwithstanding, the mapping algorithm between TCM measures and PBMs is still blank regardless of the empirical mapping research at home and abroad. The mapping algorithm presented in this study makes up for the gap, and provides a new proposal for acquiring utility value of the TCM measures.

Although the recommended mapping algorithm, showing good predictive performance, provided an approach for acquisition of utility values, there were several potential limitations in our study. First, the external validity was relatively insufficient, that is, performance evaluation of the developed mapping algorithm was still based on the whole sample; therefore, external validity needs to be assessed by utilizing external independent datasets in the future. Second, the EQ-5D-3L dataset, with inherent ceiling effect evidently, were dramatically right skewed, which led to bias and uncertainty probably; thus, a mapping algorithm between EQ-5D-5L and TCM-HSS can be explored in the future to improve the ceiling effect of EQ-5D-3L. Finally, our data were derived from single-center cross-sectional questionnaire, and it is currently unclear whether the final mapping algorithm will be affected over time; hence, a large-sample, multi-regional, and multi-center dataset can be collected in the future, and we could attempt to investigate the relationship between the mapping algorithm and time factor based on longitudinal data.

## Conclusion

It’s the first practice to develop a mapping algorithm from TCM-HSS onto EQ-5D-3L based on the direct and response mapping approaches, which exhibits excellent predictive accuracy. Thus, the utility values can be obtained from the TCM quality of life measures when EQ-5D data is not available, which supplies technical support and improve the evidence level for economic evaluation of TCM.

## Supplementary Information

**Additional file 1.** Supplementary tables and figures.

**Additional file 2. **Maps checklist.

## Data Availability

The datasets generated and/or analyzed during the current study are not publicly available due to privacy protection but are available from the corresponding author on reasonable request.

## Abbreviations

TCM-HSS: Health status scale of traditional Chinese medicine
TCM: Traditional Chinese medicine
non-PBMs: Non-preference-based measures
PBMs: Preference-based measures
EQ-5D-3L: Three-level EuroQol five-dimensional questionnaire
PE: Pharmacoeconomic evaluation
CUA: Cost-utility analysis
HU: Health utility
NICE: National Institute of Health and Clinical Excellence
OLS: Ordinary least square
CLAD: Censored least absolute deviations
GLM: Generalized linear model
ALDVMM: Adjusted limited dependent variable mixture model
TPM: Two-part model
MLR: Multinomial logistic regression
MAE: Mean absolute error
RMSE: Root mean squared error
Rho: Spearman's rank correlation coefficients
ME: Mean error
AE: Absolute error
SD: Standard deviation
