# Supplementary material for: Obtaining EQ-5D-3L utility index from the health status scale of traditional Chinese medicine (TCM-HSS) based on a mapping study
Source: Health Qual Life Outcomes. 2022 Dec 15;20:164. doi: 10.1186/s12955-022-02076-9 (PMC9753309; doi:10.1186/s12955-022-02076-9)
Supplement: Supplementary file 1 — Additional file 1. Supplementary tables and figures. [file 12955_2022_2076_MOESM1_ESM.docx]

Supplementary tables

Table S1 Comparison of the structure of the TCM-HSS and the EQ-5D measures

|  | Dimension/Subscale | Items | Levels | Total items |
| --- | --- | --- | --- | --- |
| EQ-5D-3L | mobility | 1 | 3 | 5 |
|  | self-care | 1 | 3 |  |
|  | usual activities | 1 | 3 |  |
|  | pain/discomfort | 1 | 3 |  |
|  | anxiety/depression | 1 | 3 |  |
| TCM-HSS | energy | 8 | 4 | 33 |
|  | pain | 2 | 4 |  |
|  | diet | 5 | 4 |  |
|  | stool | 3 | 4 |  |
|  | urination | 3 | 4 |  |
|  | sleeping | 3 | 4 |  |
|  | physical | 4 | 4 |  |
|  | mood | 4 | 4 |  |
|  | overall evaluation | 1 | 4 |  |

Table S2 The results of the difference test between the training and the validation dataset

|  | z | P |
| --- | --- | --- |
| Age | -0.2630 | 0.7924 |
| BMI | -0.5900 | 0.5558 |
| Gender | -0.1151 | 0.9084 |
| U | -0.1100 | 0.9156 |
| Total | 0.2110 | 0.8329 |
| EnT | 1.0230 | 0.3066 |
| PaT | 0.7980 | 0.4259 |
| DiT | 0.2910 | 0.7725 |
| StT | -1.2560 | 0.2103 |
| UrT | -1.6080 | 0.1093 |
| SlT | -0.5020 | 0.6142 |
| PhT | -0.7040 | 0.4810 |
| MoT | 0.1490 | 0.8826 |

z: z statistics; P: p-values; BMI: body mass index; U: EQ-5D-3L observed values; Total: total scores of TCM-HSS; EnT: total scores of energetic subscale; PaT: total scores of painful subscale; DiT: total scores of dietary subscale; StT: total scores of stool subscale; UrT: total scores of urination subscale; SlT: total scores of sleeping subscale; PhT: total scores of physical subscale; MoT: total scores of mood subscale;

Table S3 Spearman's correlation coefficients between EQ-5D-3L and TCM-HSS

|  | U | EnT | PaT | DiT | StT | UrT | SlT | PhT | MoT |
| --- | --- | --- | --- | --- | --- | --- | --- | --- | --- |
| U | 1 |  |  |  |  |  |  |  |  |
| EnT | -0.5073^*^ | 1 |  |  |  |  |  |  |  |
| PaT | -0.5524^*^ | 0.4962^*^ | 1 |  |  |  |  |  |  |
| DiT | -0.4582^*^ | 0.5596^*^ | 0.4274^*^ | 1 |  |  |  |  |  |
| StT | -0.3771^*^ | 0.4313^*^ | 0.2989^*^ | 0.4448^*^ | 1 |  |  |  |  |
| UrT | -0.3647^*^ | 0.3735^*^ | 0.2499^*^ | 0.4127^*^ | 0.3533^*^ | 1 |  |  |  |
| SlT | -0.3972^*^ | 0.4847^*^ | 0.4004^*^ | 0.3843^*^ | 0.3603^*^ | 0.3101^*^ | 1 |  |  |
| PhT | -0.4290^*^ | 0.4916^*^ | 0.3441^*^ | 0.4970^*^ | 0.4203^*^ | 0.3437^*^ | 0.4869^*^ | 1 |  |
| MoT | -0.4552^*^ | 0.6252^*^ | 0.4581^*^ | 0.4895^*^ | 0.3789^*^ | 0.3298^*^ | 0.5097^*^ | 0.4858^*^ | 1 |

* p < 0.05

U: EQ-5D-3L observed values; EnT: total scores of energetic subscale; PaT: total scores of painful subscale; DiT: total scores of dietary subscale; StT: total scores of stool subscale; UrT: total scores of urination subscale; SlT: total scores of sleeping subscale; PhT: total scores of physical subscale; MoT: total scores of mood subscale;

Table S4 Inter-correlations of EQ-5D-3L dimensions

|  | MO | SC | UA | PD | AD |
| --- | --- | --- | --- | --- | --- |
| MO | 1 |  |  |  |  |
| SC | 0.6543^*^ | 1 |  |  |  |
| UA | 0.7169^*^ | 0.6615^*^ | 1 |  |  |
| PD | 0.3069^*^ | 0.2729^*^ | 0.3291^*^ | 1 |  |
| AD | 0.1452^*^ | 0.1299^*^ | 0.1211^*^ | 0.2395^*^ | 1 |

* p < 0.05

MO: mobility; SC: self-care; UA: usual activities; PD: pain/discomfort; AD: anxiety/depression;

Table S5 Predicted index scores of 28 alternative models

|  | Mean | SD | Min | P5 | P25 | P50 | P75 | P95 | Max |
| --- | --- | --- | --- | --- | --- | --- | --- | --- | --- |
| U | 0.9296 | 0.1369 | 0.1140 | 0.7640 | 0.8690 | 1 | 1 | 1 | 1 |
| OLS1 | 0.9247↓ | 0.0906↓ | 0.5720↑ | 0.7522↓ | 0.8699↑ | 0.9419↓ | 1.0035↑ | 1.0225↑ | 1.0225↑ |
| OLS2 | 0.9243↓ | 0.0942↓ | 0.5621↑ | 0.7360↓ | 0.8692↑ | 0.9420↓ | 0.9954↓ | 1.0363↑ | 1.0514↑ |
| OLS3 | 0.9246↓ | 0.0924↓ | 0.5220↑ | 0.7512↓ | 0.8735↑ | 0.9513↓ | 1.0026↑ | 1.017↑ | 1.0170↑ |
| OLS4 | 0.9247↓ | 0.0940↓ | 0.5263↑ | 0.7469↓ | 0.8698↑ | 0.9490↓ | 0.9933↓ | 1.0265↑ | 1.0417↑ |
| Tobit1 | 1.1263↑ | 0.2072↑ | 0.3198↑ | 0.7320↓ | 1.0011↑ | 1.1657↑ | 1.3065↑ | 1.3500↑ | 1.3500↑ |
| Tobit2 | 1.1219↑ | 0.2126↑ | 0.2981↑ | 0.6988↓ | 0.9955↑ | 1.1566↑ | 1.2887↑ | 1.3708↑ | 1.4025↑ |
| Tobit3 | 1.1273↑ | 0.2031↑ | 0.3227↑ | 0.7580↓ | 1.0179↑ | 1.1822↑ | 1.3025↑ | 1.3407↑ | 1.3407↑ |
| Tobit4 | 1.1262↑ | 0.2069↑ | 0.3141↑ | 0.7579↓ | 0.9990↑ | 1.1664↑ | 1.2841↑ | 1.3651↑ | 1.3951↑ |
| CLAD1 | 0.9883↑ | 0.1174↓ | 0.5312↑ | 0.7648↑ | 0.9173↑ | 1.0106↑ | 1.0905↑ | 1.1151↑ | 1.1151↑ |
| CLAD2 | 0.9859↑ | 0.1139↓ | 0.5349↑ | 0.7622↓ | 0.9176↑ | 1.0066↑ | 1.0813↑ | 1.1184↑ | 1.1324↑ |
| CLAD3 | 0.9782↑ | 0.1049↓ | 0.6202↑ | 0.7614↓ | 0.9178↑ | 1.0080↑ | 1.0661↑ | 1.0862↑ | 1.0862↑ |
| CLAD4 | 1.0533↑ | 0.1714↑ | 0.2824↑ | 0.7273↓ | 0.9529↑ | 1.1099↑ | 1.1887↑ | 1.2408↑ | 1.275↑ |
| GLM1 | 0.8727↓ | 0.4257↑ | -3.5711↓ | 0.5904↓ | 0.9152↑ | 0.9676↓ | 0.9858↓ | 0.9890↓ | 0.9890↓ |
| GLM2 | 0.8528↓ | 0.5576↑ | -4.4123↓ | 0.5292↓ | 0.9173↑ | 0.9701↓ | 0.9854↓ | 0.9915↓ | 0.9927↓ |
| GLM3 | 0.8659↓ | 0.5204↑ | -4.2213↓ | 0.6112↓ | 0.9292↑ | 0.9702↓ | 0.9879↓ | 0.9900↓ | 0.9907↓ |
| GLM4 | 0.8615↓ | 0.5456↑ | -4.8383↓ | 0.6462↓ | 0.9306↑ | 0.9733↓ | 0.9869↓ | 0.9921↓ | 0.9935↓ |
| TPM1 | 0.9242↓ | 0.0877↓ | 0.5580↑ | 0.7377↓ | 0.8828↑ | 0.9621↓ | 0.9896↓ | 0.9933↓ | 0.9933↓ |
| TPM2 | 0.9236↓ | 0.0930↓ | 0.5136↑ | 0.7155↓ | 0.8845↑ | 0.9626↓ | 0.9889↓ | 0.9949↓ | 0.9963↓ |
| TPM3 | 0.9244↓ | 0.0956↓ | 0.4117↑ | 0.7363↓ | 0.8974↑ | 0.9667↓ | 0.9891↓ | 0.9921↓ | 0.9921↓ |
| TPM4 | 0.9246↓ | 0.0961↓ | 0.4062↑ | 0.7287↓ | 0.8956↑ | 0.9676↓ | 0.9880↓ | 0.9941↓ | 0.9954↓ |
| ALDVMM1 | 0.9280↓ | 0.0975↓ | 0.3981↑ | 0.7279↓ | 0.9067↑ | 0.9665↓ | 0.9882↓ | 0.9915↓ | 0.9915↓ |
| ALDVMM2 | 0.9266↓ | 0.1039↓ | 0.3729↑ | 0.6967↓ | 0.9040↑ | 0.9685↓ | 0.9873↓ | 0.9955↓ | 0.9967↓ |
| ALDVMM3 | 0.9322↑ | 0.0934↓ | 0.4007↑ | 0.7626↓ | 0.9150↑ | 0.9714↓ | 0.9892↓ | 0.9928↓ | 0.9929↓ |
| ALDVMM4 | 0.9317↑ | 0.0969↓ | 0.3851↑ | 0.7520↓ | 0.9090↑ | 0.9697↓ | 0.9891↓ | 0.9953↓ | 0.9966↓ |
| OLOGIT1 | 0.9796↑ | 0.0685↓ | 0.4690↑ | 0.7830↑ | = | = | = | = | = |
| OLOGIT2 | 0.9683↑ | 0.1102↓ | 0.1140↓ | 0.7830↑ | = | = | = | = | = |
| OLOGIT3 | 0.9605↑ | 0.1138↓ | 0.1140↓ | 0.7830↑ | = | = | = | = | = |
| OLOGIT4 | 0.9562↑ | 0.1245↓ | 0.1140↓ | 0.7830↑ | = | = | = | = | = |

U: EQ-5D-3L observed values; SD: standard deviation; OLS: ordinary least square; CLAD: Censored least absolute deviations; GLM: generalized linear model; ALDVMM: adjusted limited dependent variable mixture model; TPM: two-part model; OLOGIT: ordinal logistic regression;

Table S6 The ratio of underestimation and overestimation of alternative models

|  | Mean | SD | Min | P5 | P25 | P50 | P75 | P95 | Max |
| --- | --- | --- | --- | --- | --- | --- | --- | --- | --- |
| Overestimation | 14 | 9 | 21 | 5 | 24 | 8 | 10 | 12 | 12 |
| Percent | 50.00% | 32.14% | 75.00% | 17.86% | 85.71% | 28.57% | 35.71% | 42.86% | 42.86% |
| Underestimation | 14 | 19 | 7 | 23 | 0 | 16 | 14 | 12 | 12 |
| Percent | 50.00% | 67.86% | 25.00% | 82.14% | 0.00% | 57.14% | 50.00% | 42.86% | 42.86% |

SD: standard deviation;

Table S7 Adjustment steps for the final mapping model

|  | Step 1 | Step 2 | Step 3 | **Step 4** |
| --- | --- | --- | --- | --- |
| Dependent | 1-U | 1-U | 1-U | **1-U** |
| logit |  |  |  |  |
| EnT | 0.8095^**^ | 1.0292 | 0.7287 | **0.3972** |
| PaT | 1.4692^***^ | 0.634 | 1.6745^***^ | **1.4093^***^** |
| DiT | 0.2315 | 0.8464 | 1.4144 | **0.3034** |
| StT | 0.4311 | -0.2381 | -0.0391 | **0.0809** |
| UrT | 0.6398 | 1.8813^*^ | 1.6587 | **1.9813^**^** |
| SlT | 0.1889 | 1.087 | 0.3732 | **0.1797** |
| PhT | 1.1476^**^ | -0.1746 | 1.038 | **1.1546^**^** |
| MoT | 0.8176^**^ | 1.1387 | 1.5732^*^ | **1.6874^***^** |
| EnT2 |  | -0.1312 |  |  |
| PaT2 |  | 0.6279 |  |  |
| DiT2 |  | -0.4906 |  |  |
| StT2 |  | 0.3965 |  |  |
| UrT2 |  | -1.2365 |  |  |
| SlT2 |  | -0.5575 |  |  |
| PhT2 |  | 1.5294 |  |  |
| MoT2 |  | -0.2488 |  |  |
| EnTPaT |  |  | 0.0051 |  |
| EnTDiT |  |  | -1.6841 |  |
| EnTStT |  |  | 0.8711 | **0.7849** |
| EnTUrT |  |  | 1.6523 |  |
| EnTSlT |  |  | -0.2146 |  |
| EnTPhT |  |  | -1.0183 |  |
| EnTMoT |  |  | 0.4226 |  |
| PaTDiT |  |  | 0.5182 |  |
| PaTStT |  |  | -0.3143 |  |
| PaTUrT |  |  | -0.2237 |  |
| PaTSlT |  |  | 0.0629 |  |
| PaTPhT |  |  | -0.2312 |  |
| PaTMoT |  |  | -0.4841 |  |
| DiTStT |  |  | 0.95 |  |
| DiTUrT |  |  | -1.2786 |  |
| DiTSlT |  |  | -0.6645 |  |
| DiTPhT |  |  | 1.3247 |  |
| DiTMoT |  |  | -0.2513 |  |
| StTUrT |  |  | -2.0055^*^ | **-1.1902** |
| StTSlT |  |  | 0.7568 |  |
| StTPhT |  |  | -0.8312 |  |
| StTMoT |  |  | 0.0013 |  |
| UrTSlT |  |  | -0.3979 |  |
| UrTPhT |  |  | 3.0642 |  |
| UrTMoT |  |  | -2.3678^*^ | **-2.0671^***^** |
| SlTPhT |  |  | 0.1601 |  |
| SlTMoT |  |  | 0.1926 |  |
| PhTMoT |  |  | -0.3326 |  |
| Age |  |  |  | **0.0166^*^** |
| 1.Gender |  |  |  | **-0.2241** |
| _cons | -2.9510^***^ | -3.1005^***^ | -3.3698*** | **-3.6722***** |
| glm |  |  |  |  |
| EnT | 0.2738^*^ | -0.1689 | 0.3829 | **0.0655** |
| PaT | 0.0867 | 0.0619 | 0.0164 | **0.1072** |
| DiT | 0.1732 | 0.1151 | -0.1223 | **0.1133** |
| StT | 0.1416 | -0.0012 | -0.2922 | **-0.1838** |
| UrT | 0.3192^*^ | 0.1217 | 0.2708 | **0.2562** |
| SlT | -0.0935 | -0.4115 | -0.0562 | **-0.0592** |
| PhT | 0.0123 | 0.16 | -0.1328 | **-0.0108** |
| MoT | -0.1277 | -0.0146 | 0.0053 | **-0.0591** |
| EnT2 |  | 0.1973 |  |  |
| PaT2 |  | 0.0331 |  |  |
| DiT2 |  | -0.0145 |  |  |
| StT2 |  | 0.0849 |  |  |
| UrT2 |  | 0.1831 |  |  |
| SlT2 |  | 0.2027 |  |  |
| PhT2 |  | -0.121 |  |  |
| MoT2 |  | -0.045 |  |  |
| EnTPaT |  |  | 0.0244 |  |
| EnTDiT |  |  | 0.2573 |  |
| EnTStT |  |  | 0.4057^*^ | **0.2709** |
| EnTUrT |  |  | 0.0999 |  |
| EnTSlT |  |  | -0.33 |  |
| EnTPhT |  |  | -0.3955 |  |
| EnTMoT |  |  | -0.3558 |  |
| PaTDiT |  |  | -0.1932 |  |
| PaTStT |  |  | 0.1056 |  |
| PaTUrT |  |  | -0.205 |  |
| PaTSlT |  |  | 0.0339 |  |
| PaTPhT |  |  | 0.3002 |  |
| PaTMoT |  |  | 0.1002 |  |
| DiTStT |  |  | -0.1938 |  |
| DiTUrT |  |  | -0.0325 |  |
| DiTSlT |  |  | 0.2238 |  |
| DiTPhT |  |  | 0.1514 |  |
| DiTMoT |  |  | 0.1073 |  |
| StTUrT |  |  | 0.1613 | **-0.069** |
| StTSlT |  |  | 0.0588 |  |
| StTPhT |  |  | -0.2673 |  |
| StTMoT |  |  | -0.0426 |  |
| UrTSlT |  |  | 0.1364 |  |
| UrTPhT |  |  | -0.0153 |  |
| UrTMoT |  |  | 0.0212 | **0.0706** |
| SlTPhT |  |  | -0.0267 |  |
| SlTMoT |  |  | 0.1275 |  |
| PhTMoT |  |  | 0.2989 |  |
| Age |  |  |  | **0.0045** |
| 1.Gender |  |  |  | **-0.1093** |
| _cons | -2.0373^***^ | -1.8100^***^ | -1.9172^***^ | **-2.0378^***^** |
| N | 639 | 639 | 639 | **639** |
| MAE | 0.0605 | 0.0592 | 0.0567 | **0.05659** |
| RMSE | 0.1106 | 0.1083 | 0.1062 | **0.1039** |
| Rho | 0.6289 | 0.6379 | 0.6465 | **0.6479** |
| AE>0.05(%) | 36.31% | 34.27% | 34.27% | **34.12%** |
| AE>0.1(%) | 17.53% | 15.96% | 15.49% | **16.90%** |
| Range | 0.4317-0.9935 | 0.0058-0.9929 | -0.5944-0.9978 | **0.2567-0.9965** |
| AIC | 311.3534 | 328.1079 | 379.5479 | **305.1569** |
| BIC | 391.6316 | 479.7447 | 709.5809 | **430.0342** |

* p < 0.05, ** p < 0.01, *** p < 0.001;

U: EQ-5D-3L observed values; EnT: total scores of energetic aspect; PaT: total scores of painful aspect; DiT: total scores of dietary aspect; StT: total scores of stool aspect; UrT: total scores of pissing aspect; SlT: total scores of sleeping aspect; PhT: total scores of physical aspect; MoT: total scores of mood aspect; EnT2: EnT*EnT; EnTPaT: EnT*PaT; 1.Gender: female; N: number of samples; MAE: mean absolute error; RMSE: root mean squared error; Rho: Spearman's correlation coefficients; AE: absolute error; AIC: Akaike Information Criterion; BIC: Bayesian Information Criterion;

Table S8 The underestimation and overestimation of final mapping algorithm

|  | Overestimate(e>0) | | Underestimate e<0 | |
| --- | --- | --- | --- | --- |
|  | Frequency | Percent(%) | Frequency | Percent (%) |
| ≤0.2 | 4 | 0.63% | 0 | 0.00% |
| （0.20，0.40] | 11 | 1.72% | 0 | 0.00% |
| （0.40，0.60] | 9 | 1.41% | 0 | 0.00% |
| （0.60，0.80] | 49 | 7.67% | 14 | 2.19% |
| （0.80，1.00) | 82 | 12.83% | 45 | 7.04% |
| 1 | 0 | 0.00% | 425 | 66.51% |
| Total | 155 | 24.26% | 484 | 75.74% |

e= EQ-5D-3L predicted values - EQ-5D-3L observed values;

**Supplementary** **figures**

Figure 1 The scatter plot between EQ-5D-3L observed and predicted values


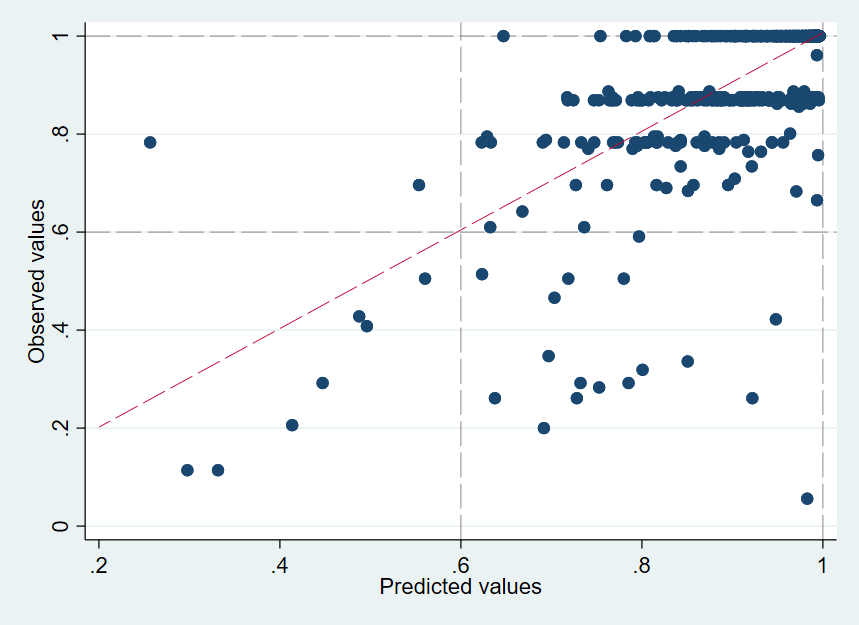


Figure 2 Line graph of MAE、RMSE、ME indicators

Figure 3 The scatter plot between EQ-5D-3L observed values and predicted error


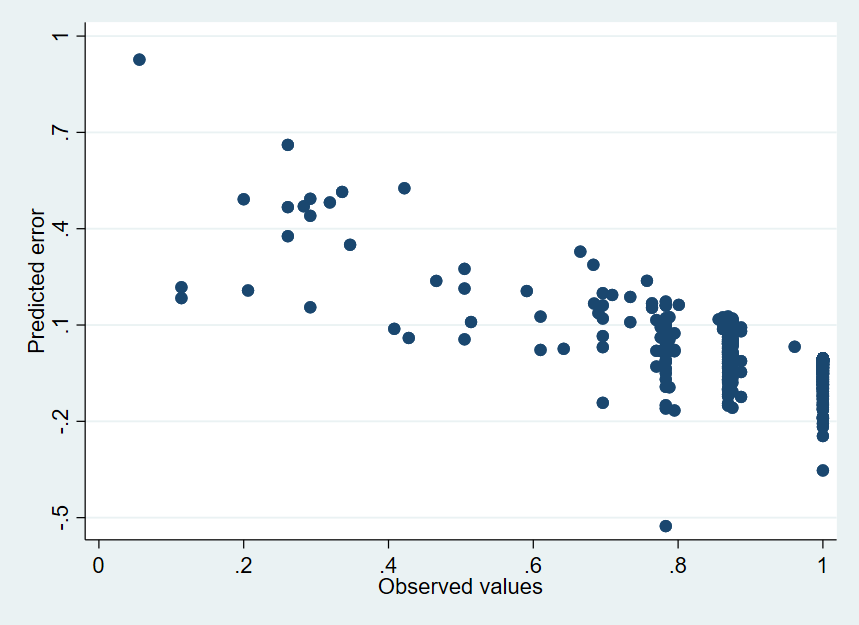


e= EQ-5D-3L predicted values - EQ-5D-3L observed values;
