# Supplementary figures and images for: Obtaining EQ-5D-3L utility index from the health status scale of traditional Chinese medicine (TCM-HSS) based on a mapping study
Source: Health Qual Life Outcomes. 2022 Dec 15;20:164. doi: 10.1186/s12955-022-02076-9 (PMC9753309; doi:10.1186/s12955-022-02076-9)

MAPS checklist

p. 5 & Table S1

p. 11-12 & Table 3

p. 7

p. 8-10 & Table 1 & 2

p. 6-7


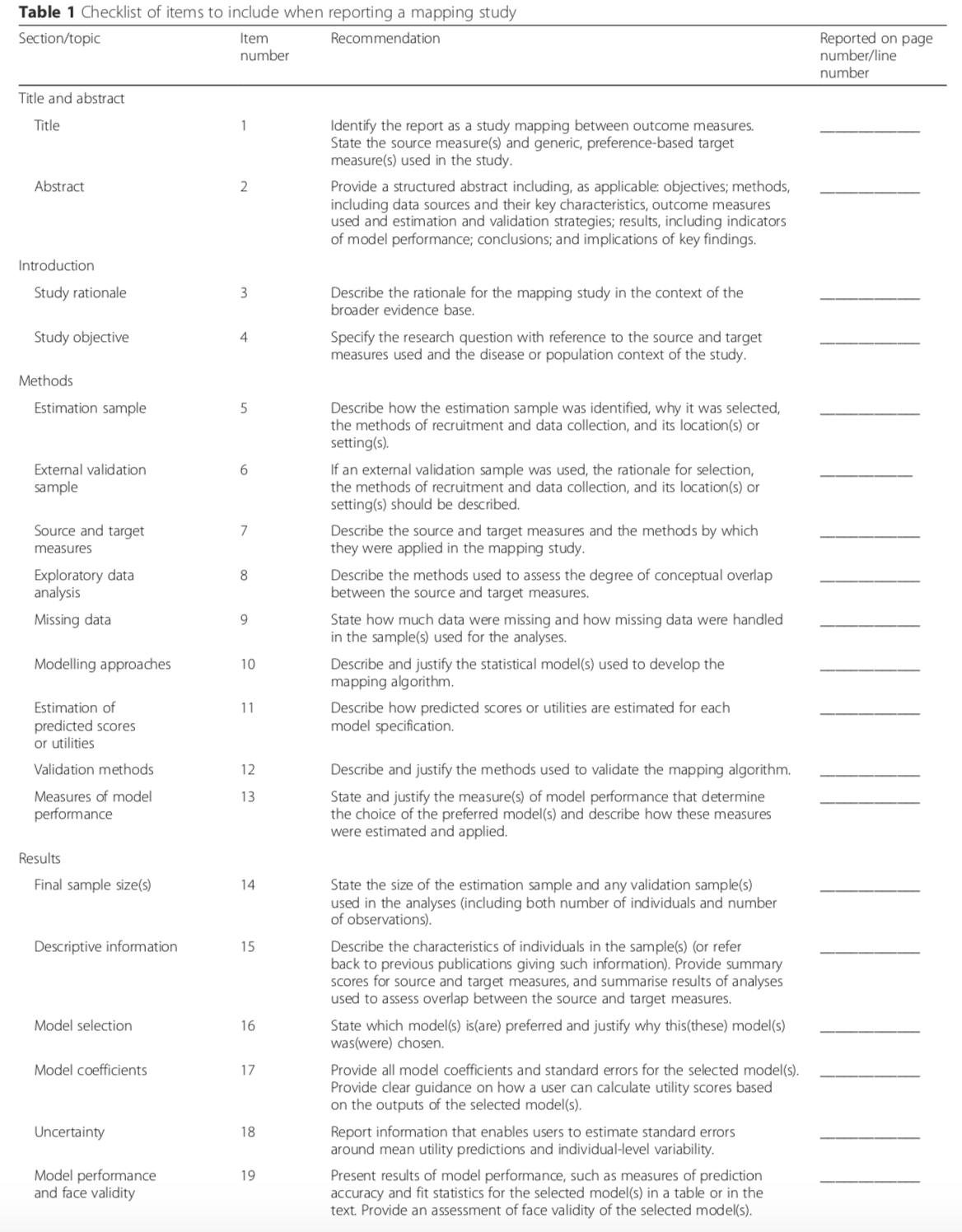


p. 1-2

p. 3-4

p. 4

p. 5-6

p. 1

p. 4 & 6

NA

p. 4

p. 7

p. 6

p. 8

Table 5 & S8

Table 4

Table 4


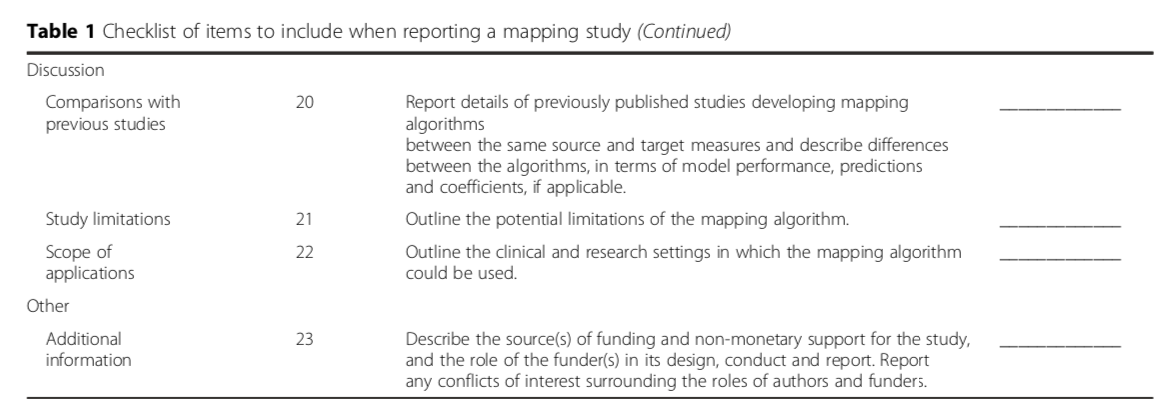


p. 3-4

p. 13-14

p. 15

p. 16-17

Supplement: Supplementary file 2 — Additional file 2. Maps checklist. [file 12955_2022_2076_MOESM2_ESM.docx]
